# Reported risky sexual practices amongst female undergraduate students in KwaZulu-Natal, South Africa

**DOI:** 10.4102/phcfm.v3i1.281

**Published:** 2011-11-10

**Authors:** Muhammad Ehsanul Hoque

**Affiliations:** 1 Department of Public Health, University of Limpopo (Medunsa Campus), South Africa

## Abstract

**Background:**

In South Africa, youths aged 15–24 years are at a higher risk of HIV infections than other age groups, and female youths are at a greater risk than their male counterparts. An essential step in controlling the pandemic of HIV and other sexually transmitted infections (STIs) is to help adolescents to reduce or avoid unsafe sexual practices.

**Objective:**

This study was designed to establish risky sexual practices amongst female undergraduate students.

**Method:**

This was a descriptive cross-sectional study carried out in September 2009 amongst full-time female undergraduate students. A multi-stage sampling method was used to recruit 391 students for the study.

**Results:**

The mean age of the students was 21.4 ± 3.2 years (range 17–45 years). More than half (52.4%) of the students were sexually active. The median age at first sexual intercourse was 19.0 years (range 12–24 years). Participants who had multiple sexual partners had a median of 2 (range, 2–4) sexual partners. The majority (89.3%) of the students used contraceptives. Almost half (41.5%), sometimes or rarely, used contraceptives during sex. With regard to substance use, 57.5% and 6.9% respectively drank alcohol and used drugs. Sexually active students had 1.5 times (OR = 1.5, *p* = 0.04), (OR = Odds Ratio), more chances of consuming alcohol than those who were not sexually active. Students with multiple sexual partners were 7 times more likely to consume alcohol compared to those who did not have multiple partners (OR = 6.9, *p* = 0.004). Students with multiple sexual partners had 3.5 times more chances of taking drugs compared to students with one steady partner (OR = 3.5, *p* = 0.038).

**Conclusion:**

A large number of female university students are engaging in risky sexual practices. University Management should concentrate on developing and implementing policies to promote safer sexual practices, in particular targeting consequences of STIs and HIV and methods to minimise the risk.

## Introduction

Sexually Transmitted Infections (STIs) pose a major public health concern globally because of their devastating impact on women and infants and their inter-relationships with HIV and AIDS. Women are exposed to the risk of unintended pregnancy and STIs over much of their reproductive lives, although consistent and correct use of contraception can reduce the likelihood of an unwanted pregnancy.^1^ A high incidence of unplanned pregnancy is indicative of unprotected sexual exposure as well as non-usage of contraceptives.^2^ Consequences of unprotected sex, such as unintended pregnancy and unsafe abortion, can be prevented through access to contraceptive services.^3^ An unintended pregnancy may force a woman to face a difficult abortion decision.^2^ A study has revealed that women who undergo induced abortion were below the age of 30 years and many of them had an above-secondary educational level.^3^ In developing countries, public health policies and programmes are focusing on the sexual and reproductive health needs of adolescents in recent years.^4^

Sexual risky behaviours are defined as sexual activities that may expose an individual to the risk of infection with HIV and other STIs. These include unprotected sex, an early sexual debut, taking alcohol or drugs before sexual intercourse, multiple sexual partners, forced or coerced sexual intercourse for reward. Lack of knowledge about HIV and AIDS and poverty have been identified as risk factors that increase the chances of young people engaging in risky sexual behaviour.^5^ When an individual who does not know his or her HIV status, engages in unprotected sex with a partner whose HIV status he or she does not know, that individual also takes the risk of being infected or infecting the partner.^6^

In South Africa, HIV prevalence amongst youths aged 15–24 is higher than in other age groups, and female youths has a higher rate (16.9%) than male youths (4.4%).^7^ There is a huge campaign to reduce the incidence of HIV through condom promotion, STI treatment programmes, the HIV and AIDS Life Skills intervention, and through mass media communications. Nevertheless, youths in South Africa continue to engage in risky sexual behavioural practices.^8^ Many youths have their first sexual experience at age 14 or younger, although the legal age of consensual sex is 16.^9^ Also, the ‘fertility conundrum’ whereby girls have to demonstrate their fertility before marriage, is evident in many African cultures, and further perpetuates the early age of sexual activity amongst youths.^8^

In South Africa, 1.5% of adult women reported to have been raped before the age of 15, and another study reported in 2003 that 15% of South African rape victims were less than 12 years old.^9, 10^ In many studies of South African secondary school pupils, 72% of pregnant teenagers, and 60% of teenagers who had never been pregnant, reported that they have had coercive sex.^11, 12^ Similarly, a study conducted in Thailand, indicated that Thai male adolescents felt that girls’ refusal to have sex was an expression of playing ‘hard to get’, and not that they did not want sex.^13^ A strong correlation was also reported to exist between gender-based violence and HIV and AIDS amongst students at two middle schools in Thailand where, even in steady dating relationships, almost half of Thai adolescents reported engaging in some form of sexual violence, and 5% had raped their girlfriends and performed anal sex.^13^

Adolescent relationships with older partners are risky because the latter often have a history of multiple relationships. Negotiating safe sex practices has diminished, and exchanging money or gifts for sex further increases the likelihood of more coercive sex.^10^ In South Africa, girls aged 15–19 years reported that they have had five partners and some of these partners were 10 years or more older than the girls.^7^ In Mexico, however, it was reported that women had partners who were 3 years older than the women.^14^

University students are at a stage in their lives characterised by searching, discovery, and experimentation, including sexual experimentation.^15^ They live and socialise with large numbers of other young adults, which encourages sexual activities that are not mutually monogamous. For this reason, university students are reportedly engaging in unsafe sex, which places them at higher risk than the general public to contract STIs, including HIV and AIDS, as well as unwanted pregnancies.^15^ Studies have shown a high engagement by students in unsafe sexual behavioural practices such as a high average number of partners, sex with unknown persons, inconsistent use of condoms, negative views about condom use, and abuse of various substances.^3, 16^ A study conducted amongst South African youths, for example, concluded that at least 50% of young people are sexually active by the age of 16, and 50%–60% of sexually active youths report that they never use condoms.^6^ Another study found that, in South Africa, about 75% of unplanned pregnancies occurred amongst unmarried women.^17^ Studies have also identified substance abuse as a risk for sexual risk taking.^18, 19, 20^

The National HIV, AIDS and STI Strategic Plan for South Africa 2007–2011 has two primary aims. They are to reduce the number of new infections by half, and to reduce the impact of HIV and AIDS on individuals, families and communities. With respect to sectoral HIV and AIDS interventions, the Department of Education (DoE) has also implemented programmes with objectives aligned to the National Strategic Plan. At a sub-sector level, the Higher Education HIV and AIDS Programme (HEAIDS) strategic framework reflects the strategic objectives of the sector. It has aligned its key result areas along the national, and sector HIV and AIDS strategies. HEAIDS coordinates HIV and AIDS programmes at all institutions of higher learning in the country, with a view of to strengthen capacity and to respond comprehensively to the related challenges. In line with the national HEAIDS policy, the campus HIV and AIDS programme exists to reduce the threat of the spread of HIV and AIDS amongst students, staff and the surrounding community and to mitigate the impact thereof.

An essential step in controlling the pandemic of HIV and STIs is through helping adolescents to reduce or avoid unsafe sexual behaviour. The provision to adolescents of the information, motivation, and interpersonal skills needed to avoid sexual risk (e.g. to abstain) and to reduce risk (e.g. condom use), is an important aspect of reducing the spread of HIV. Thus, the prevention of HIV and STIs in university settings is important.

At present, there is no programme designed to promote and encourage safe sex behaviour in the University community, and hence this study was aimed at documenting the pattern of sexual behaviour amongst female students of Mangosuthu University of Technology (MUT). Findings of the study may serve as a platform for developing interventions for the promotion of safer sexual behaviour and practices amongst students in the University.

### Significance of the study

A large number of female university students engage in risky sexual behaviour as well as in substance use. There is currently no programme in the University that promote healthy sexual practices amongst students. University Management needs to concentrate on developing and implementing policies on health education and promotion, particularly targeting consequences of STIs and HIV and how the risk could be minimised.

## Ethical considerations

Ethical permission for the study was obtained from the Ethics task team of the Faculty of Natural Sciences Research and Publications Committee of MUT. Informed consent from participants was also obtained. Confidentiality of participants was maintained at all times. No form of identifiers was included in the questionnaires to maintain confidentiality further. Participation was voluntary and participants were informed that they could withdraw from the study at any stage of the interview if they so desired, without any penalty.

## Methods

### Setting

Mangosuthu University of Technology (MUT) is located on Umlazi Highway in Umlazi, about 18 kilometers south of the busiest port city (Durban) in KwaZulu-Natal (KZN) province of South Africa. The KwaZulu-Natal province had the highest HIV prevalence rate of 15.8% in South Africa in 2008.^21^ The university has a unique setting within a sub-rural area, once ‘township’, called Umlazi in the now Emawaleni District of KZN, which is populated by predominantly historically disadvantaged people of South Africa. Its programmes therefore give priority to students from historically disadvantaged communities. In 2009, the student population for day students was 8345, of which 4244 were female and 4101 were male students. The University offers postgraduate qualifications and national diplomas with academic teaching during both the day and evenings. There are three Faculties namely, Natural Sciences, Engineering and Management Sciences.

### Study design, sampling, and data collection

This was a descriptive cross-sectional study carried out in September 2009 amongst full-time female undergraduate students. A sample of 391 students was selected based on 95% confidence level, and statistical power of the study as 90%. Samples were selected by multi-stage sampling methods. Firstly, stratified random sampling technique was used where different faculties of the University were considered as strata. Then, from each faculty, numbers of students were selected based on probability proportional to size (PPS) sampling technique. The study was conducted by means of a questionnaire survey. The questionnaire included questions on behavioural profile, and sexual behaviour. Questions were simple and concise. The questionnaire was pretested on 10 female students from an extended programme, who were not part of the study, to identify gaps and modify the questionnaire appropriately.

The researcher allocated dates for different faculties. Prior to the dates, the researcher selected classes randomly and then communicated with the corresponding lecturer about the data collection procedure. Once the lecturer agreed, the researcher went to the corresponding lecturer's lecture room and attended the lecture. Once the lecturer finished his or her lecture (the lecture finished 20 minutes before the scheduled time as agreed upon with the researcher), the lecturer introduced the researcher to the class. The researcher then provided an explanation of the study including the purpose and objectives. The participants were also informed that the study was voluntary and confidential. A written informed consent was signed by every participant and the questionnaire was completed in the presence of the researcher within 20 minutes.

### Analysing

Data were entered into Microsoft Excel 2003 spreadsheet and imported to SPSS 17.0.1 Window version for analysis. The results of participants’ were summarised using descriptive summary measures expressed as mean (s.d.) or median (range) for continuous variables and percentage for categorical variables. Chi-square test was used to find associations between categorical variables. Odds ratios (OR) with 95% confidence intervals were also calculated. All statistical tests were performed by using two-tailed tests at the 0.05 level of significance. *P*-values less than 0.05 were considered statistically significant.

## Results

A total of 391 students completed the self-administered questionnaire. The mean age of the students was 21.39 ± 3.15 years (range, 17–45 years). Almost all (95.7%) of the students were single and the rest (4.3%) were married or divorced. Amongst the students, 40.4% were first year students followed by second year (41.7%) and third year (17.9%) students.

Sexual practices of the participants have been summarised (Table 1). Of the respondents, 52.4% of the female students were sexually active. Amongst the sexually active students, 89.3% had an ongoing sexual relationship with a single partner. The median age at first sexual intercourse was 19.0 years (range, 12–24 years). About one-tenth of respondents reported multiple sexual partners with a median of two (range, 2–4) sexual partners during the previous 12 months. Almost a fifth (18%) of the sexually active students reported an STI diagnosed during the previous 12 months.

**TABLE 1:** Sexual practices of the participants.

Variables	Frequency		%
**Presently sexually active (*n* = 391)**			
Yes	205		52.4
No	186		47.6
Median age of first sexual intercourse (range, years)		19 (12–24)	
**Have multiple partners (*n* = 205)**			
Yes	22		10.7
No	183		89.3
Median no of multiple partners in the last 12 months (range) (*n* = 22)		2 (2–4)	
Treated for STIs (*n* = 205)	37		18.0
Consumed alcohol (*n* = 391)	225		57.5
Sexual intercourse under the influence of alcohol (*n* = 225)	30		13.3
Used drugs (*n* = 391)	27		6.9
Sexual intercourse under the influence of drugs (*n* = 27)	2		7.4

*Source*: Author's original data

With regard to contraceptive uses, the majority (89.3%) of the respondents used contraception. Condoms were the main method of contraception; 82.5% used it. More than half (58.5%) of the respondents always used contraceptives during sexual intercourse (Table 2). Amongst those who did not use contraception, 11 students indicated unavailability of contraceptives as the reason and only 5 students mentioned that the partner did not want it.

**TABLE 2:** Contraceptive practices amongst sexually active students.

Variables	Frequency	%
**Contraceptive use (*n* = 205)**		
Yes	183	89.3
No	22	10.7
**Reasons for not using contraceptives**		
Unavailability of contraceptives	11	50.0
Lack of knowledge about contraceptives	5	22.7
Partner did not want it	5	22.7
Did not think of contraceptive at the time	1	4.6
**Used contraceptives during last sexual intercourse (*n* = 183)**		
Yes	145	79.2
No	38	20.8
**Contraceptive methods (*n* = 183)**		
Condom (woman or her partner)	151	82.5
Contraceptive pills	20	10.9
Injectables	6	3.3
Withdrawal	4	2.2
Safe period	2	1.1
**How often are contraceptives used (*n* = 183)**		
Always	107	58.5
Sometimes	65	35.5
Rarely	11	6.0

*Source*: Author's original data

The association between substance use and other variables has been analysed (Table 3). With regard to substance use, 57.5% consumed alcohol and 6.9% used drugs. Significantly, more sexually active (62.4%) versus not sexually active (52.2%) students drank alcohol, which indicates that sexually active students had 1.5 times (OR = 1.5, 95% CI: 1.0–2.3, *p* = 0.04) more chances of consuming alcohol than those who were not sexually active. There was found also a significant association between multiple sexual partners and the consumption of alcohol, because students who had multiple sexual partners were 7 times more likely to drink alcohol, compared to those who did not have multiple partners (OR = 6.9, 95% CI: 1.6–30.6, *p* = 0.004). Age (20 years or less vs. more than 20 years) was not associated with drinking alcohol (*p* = 0.278). Use of drugs was significantly associated with age because students more than 20 years old were 4.41 times more likely to use drugs, compared to those who were below the age of 21 (OR = 4.41, 95% CI: 1.495–13.010, *p* = 0.004). Furthermore, students with multiple sexual partners had 3.475 times more chance of taking drugs, compared to students with one steady partner (OR = 3.475, 95% CI: 1.002–12.045, *p* = 0.038).

**TABLE 3a:** Substance use amongst the participants.

Variables	Consumed alcohol		*p*-value
		
	No	Yes	
**Age group (years)**			
20s or younger	74	88	0.278
Older than 20	92	137	
**Presently sexually active**			
No	89	97	0.040
Yes	77	128	
**Multiple sexual partners**			
No	75	108	0.004
Yes	2	20	

*Source*: Author's original data

**TABLE 3b:** Substance use amongst the participants.

Variables	Took drugs		*p*-value
		
	No	Yes	
**Age group**			
20 years or younger	158	4	0.004
Older than 20	206	23	
**Presently sexually active**			
No	174	12	0.736
Yes	190	15	
**Multiple sexual partners**			
No	172	11	0.038
Yes	18	4	

*Source*: Author's original data

## Discussion

The study was carried out to establish the sexual behaviour amongst female university students in South Africa. The data indicated that 52.4% of students were sexually active at the time of the study. This finding is similar to a study conducted amongst undergraduate students in Nigeria (54%), but much lower than the findings in other African countries such as Madagascar (80%).^22, 23^ This study only included female students, whereas the other studies included male as well as female students, and male students are also more sexually active than female students. In addition, HIV and AIDS campaigns in the country, as well as in this institution, may have played a role in (some) students abstaining from sex despite the fact that they were at the age of sexual interest and experimentation.

The age of sexual debut and the number of sexual partners are the most important factors related to the acquisition of STIs and HIV. In the present study, the median age and number of sexual partners was 19 years and 2, respectively, in the previous 12 months. This is consistent with results from Madagascar and Brazil.^23, 24^ A review study amongst South African women found that the median age of sexual debut was 17 years and the median number of sexual partners was 2.^25^ A study conducted amongst Wentworth pupils reported that 9.6% of female pupils had their first sexual experience before age 12, with a higher prevalence between 12 and 15 years. This high-risk behaviour was also reported amongst secondary school pupils in Cape Town, where 10.8% of female pupils were sexually active in grade 8.^26^ Adolescents who begin sexual activity early are more likely to have more sexual partners, and therefore are exposed more to the risk of HIV. Another study reported that urbanisation, poverty, exposure to conflicting ideas about sexual values and behaviour, and encouraging premarital sexual activity amongst adolescents, may be factors that contribute to early sexual initiation of young people globally.^27^ A recent survey amongst adolescents in KwaZulu-Natal suggested that receiving ‘health shocks’, for example the death of a loved one, are risk factors for sexual debut. Adolescents in a household that experienced a recent death were 20% more likely to have debuted sexually, whilst those who experienced a recent severe illness were 13% more likely to have debuted sexually. The Wentworth pupils might therefore have experienced emotional shocks as a result of illness or death within their family and social circles, which in turn inclined them to be more sexually active and to ignore the risk of contracting HIV and AIDS.^28^

Engaging in multiple sexual partner relationships is considered one of the higher risk sexual behaviours responsible for the spread of STI, HIV and AIDS. In this study, one in ten female students was involved in multiple sexual relationships. In another study it was suggested that poverty and the need to survive sometimes force women into risky sexual behaviour, for example, prostitution and multiple partners.^29^

The majority of the sexually active students used contraception and condom use was the main method of contraception, followed by contraceptive pills. This finding is similar to other studies conducted in South Africa.^30, 31, 32^ In Ethiopia, amongst female university students, 10% of the respondents claimed to have used contraceptive methods, whereas a study in Nigeria found that 30.1% had used a contraceptive method at some stage.^3, 33^ Condom use is promoted globally as an effective means of reducing sexual risk, more specifically HIV and sexually transmitted infections. For HIV and AIDS behaviour-change-communication programmes to change behaviour they need to be acceptable and accessible and they have to yield the desired outcomes. Condoms are widely distributed at other community settings and students could access them from these settings, which may explain the low rate of condom distribution on campus.

Research into behaviour and beliefs of adolescents about condom use, suggests that inconsistent condom use is more of the norm than the exception amongst sexually reproductive adults.^34^ In this study, 41.5% of sexually active female students mentioned that they used contraception sometimes or rarely. In terms of how often contraceptives were used, the study from South Africa found that only 22.5% of female students always used contraceptives.^17^ The utilisation of contraceptives by the female Lebanese undergraduate students has been reported as low.^1^ The study conducted in Ethiopia amongst the university students also showed that about 4.9% of the students have used contraceptives and that the pill (44%) was the most commonly used method, followed by the injectables (13%).^3^ The study in Botswana reported that it is far more unlikely for teenagers to use contraception than it is for older women, and that the most popular method is the pill.^2^ This trend is not limited to sub-Saharan Africa. Amongst sexually experienced Slovakian students, for example, it was reported that inconsistent condom use was the most prevalent risk behaviour in both genders, at 81% and 72% in female and male students, respectively.^35^ It is likely, therefore, that condom use in this high-risk group is inconsistent.

With regard to STIs, 18% of the sexually active students reported to have been treated for an STI. A South African national survey reported that 23% of the young male and female students aged 15–24 years had been treated for an STI at some point in their lives, and this is indicative of inconsistent condom use.^21^ This result is higher than in a study conducted amongst Brazilian and American undergraduate students.^24, 36^

Another behavioural risk concerns the use of alcohol and drugs before sex. It reduces a person's cognitive ability to consider protective sex, and increases susceptibility to coercive sex.^37^ In this study, 13.3% students admitted to sex under the influence of alcohol. This finding is lower than that of the study conducted amongst French students (37%).^38^ This study also reported that there was an association amongst students between taking drugs (6.9%) and multiple sexual partners. Cannabis use was found to be associated with multiple sexual partners.^39, 40^ A Spanish study, for example, that considered a variety of drugs and sexual behaviour, found that a greater frequency of drunkenness and cannabis use were related with having more than one sexual partner, sexual risk behaviour (i.e. more than one partner and failure to use a condom regularly) was more frequent amongst persons who had been drunk, or used cannabis or cocaine.^41^ A study conducted amongst young people indicated an inconsistent use of condoms amongst PLWHA (People Living with HIV and AIDS) with alcohol problems, and it was less likely that a condom would be used when substance use preceded sexual intercourse with a person who was not their partner.^42^ There was also a correlation between the consumption of alcohol and multiple partners. Researchers further reported that alcohol and drug use did not influence unprotected sex; this conforms to other studies which found alcohol and drug use to be inconsistently related to protective behaviours (e.g., condom use).^34, 43^ It was reported in another study that alcohol use in conjunction with sex is not related to condom use amongst men and women. There is a close correlation, however, between drug use and non-use of condoms, and it is argued further that alcohol use is associated with sexual risk behaviours amongst drug users.^44^

### Limitations of the study

There were numerous limitations that could arise from the study methodology. The study population consisted of students at one university, and it might not be advisable to generalise our results with regard to other universities. Students’ behaviours were self-reported. In addition, although the surveys were anonymous, some students may have been reluctant to report sensitive information regarding sexual behaviour, which would have led to underestimates of risky behaviours. To minimise this type of bias, confidentiality was ensured.

## Conclusion

Safe sexual behaviour is very important to a student's short-term and long-term reproductive health. A large number of female students in this study engage in risky sexual practices; thus it is necessary to modify social and educational activities to improve understanding of the consequences of unsafe sexual behaviour and how the risk can be minimised.
